# The Ocular Surface Characteristics in Prostate Cancer Patients Treated with Androgen Deprivation Therapy

**DOI:** 10.1155/2021/5390195

**Published:** 2021-11-09

**Authors:** Weina Li, Xiaofeng Li, Feilun Cui, Zhipeng Xu, Nuo Dong, Cheng Li

**Affiliations:** ^1^Eye Institute & Affiliated Xiamen Eye Center, School of Medicine, Xiamen University, Xiamen, China; ^2^Fujian Provincial Key Laboratory of Ophthalmology and Visual Science & Ocular Surface and Corneal Diseases, Xiamen, China; ^3^Department of Ophthalmology, Changhai Hospital, Shanghai, China; ^4^Department of Urology, Zhenjiang First People's Hospital, Zhenjiang, China

## Abstract

**Background:**

To investigate the association of long-term androgen deprivation therapy (ADT) with ocular surface characteristics in prostate cancer patients.

**Methods:**

A total of 30 male prostate cancer patients who received ADT were selected. All candidates were scored using the Ocular Surface Disease Index (OSDI) and subsequently divided into two groups containing 9 symptomatic patients (scores >12) and 21 asymptomatic patients (scores ≤ 12). Another 20 healthy age-matched males were selected as the control group. Each candidate was assessed with respect to eyelid margin abnormality, tear film break-up time (NI-BUT), tear meniscus height (TMH), meiboscore, meibum expressibility, and demodex infection.

**Results:**

The NI-BUT in the ADT group was significantly shorter than that in the control group. The scores for OSDI, eyelid margin abnormality, meibum expressibility, and meiboscores were significantly higher in the ADT group (*P* < 0.05). Moreover, the NI-BUT in the symptomatic ADT group was significantly shorter than that in the asymptomatic ADT group (*P* < 0.05). The meiboscores and meibum expressibility score in the symptomatic ADT group were significantly higher than those in the asymptomatic ADT group (*P* < 0.05). The presence of demodex in the symptomatic ADT group was also higher than that in the asymptomatic ADT group (*P* < 0.05).The length of time that patients had been taking ADT was positively correlated with meiboscores and negatively correlated with NI-BUT.

**Conclusion:**

Androgen levels were associated with significant changes in relative meibomian gland function. Subjective symptoms, such as dryness and foreign body sensation, were more obvious in prostate cancer patients receiving ADT, which may be caused by MGD and demodex infection. It's recommended that more attention be paid to the ocular surface in prostate cancer patients taking ADT by performing examination of NI-BUT and meibomian gland morphology and function with a view to providing more comprehensive prevention and treatment protocols.

## 1. Introduction

Prostate cancer is the most common malignant tumor in men, and its incidence is gradually increasing annually. As early as 1941, Huggins and Hodges proposed androgen deprivation as an effective treatment for prostate cancer [1]. Nowadays, androgen deprivation therapy (ADT) is the mainstay of treatment for locally recurrent or advanced prostate cancer [2]. Although ADT is customized based on individual disease characteristics, side effects of these drugs are inevitable due to the long-term lack of androgen regulation. The effect of meibomian gland damage on the development of dry eye after long-term ADT has rarely been evaluated.

The meibomian glands can synthesize and secrete lipids to form the superficial tear film layer. Meibomian gland dysfunction (MGD) is a chronic diffuse abnormality of the meibomian glands, characterized by terminal duct obstruction and/or qualitative/quantitative changes in glandular secretion [3]. MGD is thought to be caused by abnormal lipid excretion that results in increased evaporation, leading to evaporative dry eye disease (DED). Previous studies have shown that androgens can exert a protective function on the ocular surface [4].

The meibomian glands are an androgen target. They express androgen receptor proteins in acinar epithelial cell nuclei [5], in addition to mRNAs for type 1 and/or type 2 5*α*-reductase, which convert testosterone to the very potent dihydrotestosterone [6].

Androgens play a key role in meibomian gland physiology, stimulating function and suppressing keratinization [7]. Schirra et al. [8] demonstrated that androgens regulate the expression of more than 1590 genes involved mainly in lipid metabolism in the mouse meibomian gland. Moreover, androgen deficiency is a risk factor for MGD and evaporative DED [7, 9]. MGD and DED have been reported to be associated with age and gender, the prevalence of which is higher in the elderly and in women [7, 10]. Although there is some evidence supporting the ability of chronic androgen deficiency to alter lipid secretion in meibomian glands, leading to MGD and DED [11], there is no clinical confirmation of their relationship with symptoms of dry eye or treatment time in patients taking androgen deprivation drugs.

To date, there have been only limited investigations regarding the possible association between androgens and meibomian glands. Given this background, we predict that androgen deficiency will influence meibomian gland function, alter meibum secretion, and decrease tear film stability, and that abnormality of the ocular surface in prostate cancer patients is related to the length of time taking ADT. To test this hypothesis, we sought to determine the effects of androgen deficiency on the ocular surface and meibomian glands by comparing prostate cancer patients receiving long-term ADT with age-matched male controls.

## 2. Materials and Methods

The study sample comprised 30 male prostate cancer patients (74.77 ± 8.62 years old, *n* = 60) receiving long-term ADT and 20 age-matched male controls (72.30 ± 6.19 years old, *n* = 40) who had not received ADT. The average duration of ADT in prostate cancer patients was 15.06 months (range 3–60 months). For the ADT group, prostate cancer patients receiving ADT was the sole inclusion criterion. The exclusion criteria were infectious keratitis or conjunctivitis, continuous eye drop use, history of eye surgery, and serious systematic disease. All participants were recruited from Zhenjiang First People's Hospital between June 2018 and December 2019.

### 2.1. Symptom Assessment

Dry eye symptoms were assessed using the OSDI Questionnaire, which comprises 12 questions regarding the frequency of ocular discomfort, visual problems interfering with daily activity, and ocular discomfort caused by environmental factors [12]. Total OSDI was calculated using the following formula: OSDI = [(sum of all answered questions) × 100]/[total number of answered questions) × 4]. Total scores were expressed on a scale of 0 to 100, with higher scores representing a greater disability [13]. Subjects with a score > 12 were placed into the symptomatic group, and the rest were placed into the asymptomatic group.

### 2.2. Noninvasive Tear Film Break-Up Time

All subjects underwent imaging with a Keratograph (Oculus, Wetzlar, Germany) that measured the noninvasive tear film breakup time (NI-BUT). Subjects focused on the central target, and a Placido disc pattern comprising 22 illuminated rings was projected onto the corneal surface. Subjects were asked to blink twice and keeped their eyes open until the next eye blink or the sampling procedure ended. Then, the first NI-BUT was recorded.

### 2.3. Tear Meniscus Height

The tear meniscus height (TMH) was evaluated using a Keratograph that had four infrared diodes under deactivation of red ring illumination and measured perpendicular to the eyelid margin at the 6 o'clock position of the corneal midline [14]. All examinations were performed three times.

### 2.4. Meibographic Evaluation

Meibography was accomplished using the infrared camera system of the oculus Keratograph. The meibomian glands were visualized by everting the eyelid. Areas of meibomian gland dropout and total tarsal plate were measured to calculate the percentage of meibomian gland dropout. Meiboscores were assessed according to the grading scale as described by Arita et al. [15]: grade 0, no loss of meibomian glands; grade 1, area loss < 33% of the total meibomian gland area; grade 2, area loss between 33% and 67% of the total meibomian gland area; and grade 3, area loss > 67% of the total meibomian gland area.

### 2.5. Eyelid Margin Abnormality

Under a slit lamp microscope, eyelid margin abnormality was quantitated as the total score of the following 4 parameters: plugged meibomian gland orifices, eyelid margin irregularity, eyelid margin vascular engorgement, and mucocutaneous junction displacement. The score ranged from 0 to 4 depending on the number of abnormalities present [16].

### 2.6. Examination of Demodex Infection

A total of 4 eyelashes from each eyelid were removed using fine forceps and placed on a glass slide. A drop of 70% glycerinum was added to the lashes before a coverslip was mounted. Under a light microscope, demodex was identified based on morphology and movement.

### 2.7. Meiboscores

After placing a hot compress on the eyelid for 10 minutes, pressure was applied to the upper and lower eyelid using two cotton buds to encourage the meibomian glands to secrete lipids. Characteristics of the meibum were graded on a numerical scale of 0–3 as follows [17]: grade 0, clear fluid; grade 1, cloudy fluid; grade 2, cloudy fluid with granular debris; and grade 3, thick toothpaste-like fluid. The higher the meiboscore, the cloudier and more viscous the meibum.

### 2.8. Statistical Analysis

The data were analyzed using SPSS 26.0 (Inc, Chicago, Illinois, USA) and are expressed as the mean ± standard deviation. Comparison of clinical parameters between the ADT and control groups was performed using the Mann–Whitney *U* test, *X*^2^ test, and Fisher's exact probability test. Correlations between clinical parameters in the ADT group were evaluated by Spearman's correlation. *P* < 0.05 was considered statistically significant.

## 3. Results

A total of 100 eyes (ADT group, 60; control group, 40) were evaluated. Subjects in both groups were age-matched elderly males. The reference value for serum testosterone in healthy men was 1.75–7.81 ng/mg. This study selected prostate cancer patients whose serum testosterone value was 0.10 ± 0.13 ng/mg, which is significantly below normal.

**Figure 1:**
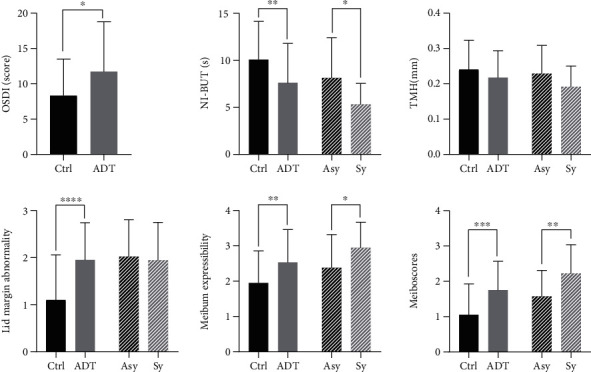
Mann–Whitney *U* test of each examined item between prostate cancer patients receiving ADT and the control group, as well as between the asymptomatic and symptomatic ADT groups (^∗∗∗^*P* < 0.001, ^∗∗^*P* < 0.01, ^∗^*P* < 0.05).

The dry eye symptoms in prostate cancer patients receiving long-term ADT were more severe than those in control individuals (Figure 1). In comparison with the control group, the ADT group exhibited a significantly shorter NI-BUT (*P* = 0.004; Table 1). The OSDI, eyelid margin abnormality score, and mean meiboscores as well as meibum expressibility scores in the ADT group were significantly higher than those in the control group (all, *P* < 0.05; Table 1); however, there was no significant difference in TMH between the two groups.

According to the OSDI scores, prostate cancer patients were divided into the symptomatic ADT group (scores > 12) containing 9 patients and the asymptomatic ADT group (scores ≤ 12) containing 21 patients. Following subgroup analysis of patients with established dry eye symptoms and those without, we found that the NI-BUT in the symptomatic ADT group was significantly shorter than that in the asymptomatic ADT group (*P* < 0.05). The mean meiboscores and meibum expressibility scores in the symptomatic ADT group were significantly higher than those in the asymptomatic ADT group (*P* < 0.05). Ocular surface discomfort was associated with a decrease in NI-BUT. There were no significant differences in the eyelid margin abnormality score or TMH between the symptomatic and asymptomatic ADT groups according to the completion of a questionnaire designed to assess dry eye symptoms.

Previous research has shown that demodex infestation is closely related to meibomian gland dysfunction and ocular surface inflammation [18]. In the present research, the percentage of demodex infection was 60.0% and 70.0% in the ADT and control group, respectively. There was no significant difference in demodex infection rate between these two groups (*P* > 0.05; Table 2). However, following grouping of the prostate cancer patients based on OSDI score, all 9 symptomatic patients had demodex infection. For the symptomatic and asymptomatic groups, the demodex infection rate was 100% and 57.14%, respectively. The difference was statistically significant (*P* < 0.05; Table 2).

MGD is a common and chronic progressive disease that has an adverse impact on the ocular surface. The duration of ADT as a time-dependent variable was examined for its association with MGD. In the present study, correlation analysis between clinical parameters in the ADT group (Table 3) revealed that the treatment time was positively correlated with the meiboscores and negatively correlated with the NI-BUT.

## 4. Discussion

Androgens are known to induce the proliferation and differentiation of sebaceous glands throughout the body [19]. Given that the meibomian glands are large sebaceous glands that express androgen receptors, androgens also play an important role in meibomian gland development. However, long-term ADT suppresses androgen levels, which can slow or stop the growth of prostate cancer, but this leads to physiological changes in the ocular surface. The present study investigated the changes in the structure and function of the ocular surface in prostate cancer patients taking ADT. These patients experienced a greater frequency of tear film debris, irregular eyelid margins, atrophy and orifice metaplasia of the meibomian glands, and a marked decrease in the quality of meibomian gland secretions than age-matched controls.

Dry eye syndrome is a relatively common. Analysis of the OSDI scores results indicated that ADT group did have a higher frequency of dry eye syndrome. Tear film stability is evaluated by measuring the NI-BUT. There was a significant decrease in NI-BUT values in the ADT group as compared with those in the control group, demonstrating a decrease in tear film stability. The eyelid margin abnormality, meibum expressibility, and meiboscores were higher than those in the control group, indicating changes in the morphology and function of meibomian glands in patients taking ADT. Androgens have been shown to significantly affect lipid metabolic pathways [20], and the synthesis and/or elaboration of lipids by the meibomian glands is thought to be regulated by androgens [5]. A previous study showed that ADT can exert a significant and consistent influence on the mass/charge ratios of neutral lipid fraction of meibomian gland secretions [21]. Moreover, Jennifer et al. [22] reported that androgen insensitivity can cause meibomian gland dysfunction and significantly increase dry eye signs and symptoms in patients with complete androgen insensitivity syndrome (CAIS). Androgens can also modulate the morphology, physiology, molecular biology, immunology, and secretion of the lacrimal gland [23]. Interestingly, Sullivan et al. [24] demonstrated that ADT has no influence on the level of tear secretion in humans. Similarly, no significant difference in TMH between the two groups was found in the present study. Androgen deficiency appears to cause the development of meibomian gland dysfunction and evaporative dry eye.

Prostate cancer can be aggressive and metastasize to the bones and regional lymph nodes. There are no reports of metastasis to the eye; therefore, prostate cancer itself does not cause tear function-related damage to the ocular surface. To further investigate the reasons for dry eye in prostate cancer patients treated with ADT, we divided these patients into symptomatic and asymptomatic subgroups based on the OSDI score. Our results indicate that the NI-BUT was shorter in the symptomatic ADT group than that in the asymptomatic ADT group. A significant difference was noted in meibum expressibility scores and meiboscores between these two groups (*P* < 0.05 for each), indicating that the tear film stability was worse in the symptomatic ADT group. Meibomian glands secrete lipids into the tear film, forming a superficial lipid layer that can maintain tear film stability. It can be concluded that the reason for tear film instability in the ADT group, especially in those with dry eye symptoms, may be abnormalities in the meibomian glands.

Demodex mites are a potential cause of MGD [25]. The demodex infestation rate was significantly higher in the symptomatic ADT group (9/9, 100.00%, Table 2) than that in the asymptomatic ADT group, indicating that the ocular discomfort experienced by prostate cancer patients was related to ocular demodex infestation. Franz et al. [26] reported that the prevalence of demodex in patients with ocular discomfort was high. Moreover, Lee et al. [27] showed that the number of demodex mites was proportional to the OSDI score and the severity of ocular discomfort. Taken together, this evidence implies that demodex infestation can cause an increase in the subjective symptoms of ocular surface damage. Thus, eradication of demodex may be beneficial in relieving ocular surface discomfort related to MGD.

MGD has many underlying causative factors such as age, gender, and hormones. Aging may impact atrophy of meibomian gland acini epithelial cells and reduce the quality of meibomian gland secretions [28]. Decreased sex hormone levels, especially androgens, which are correlated with advancing age, may contribute to meibomian gland loss [24], leading to increased viscosity of the meibum, hyperkeratinization of the meibomian gland orifices, and meibomian gland obstruction [29]. MGD due to androgen deficiency has been demonstrated in prostate cancer patients receiving ADT [11]; however, no studies to date have reported an association of ADT duration with changes in the ocular surface. In the present study, the duration of ADT was positively associated with the meibomian gland dropout score in the ADT group but negatively correlated with the NI-BUT. This indicates that a longer duration of ADT was associated with poorer tear function and a larger meibomian gland dropout area. Similarly, decreased sebaceous gland function and secretion are correlated with both an atrophy of acinar cells and a reduction in serum androgen levels [30]. Meibomian gland atrophy tends to increase with age, and a significant correlation between age and loss of meibomian gland area was observed by infrared meibography in a previous study [14]. In short, the androgen level is associated with meibomian gland alterations. Thus, we speculated that androgens may stimulate meibomian gland function, enhancing tear film stability and decreasing tear film evaporation and consequently ameliorating dry eye symptoms. A longer duration of ADT can lead to a significant increase in the risk of MGD.

To the best of our knowledge, this is the first study to investigate the association between long-term ADT and the risk of MGD in an Asian population. Our study focused on the relationship between androgens and the ocular surface and found that androgen deficiency was related to ocular discomfort, eyelid margin abnormality, and meibomian gland loss. In conclusion, we demonstrate that long-term ADT in prostate cancer patients increases the risk of MGD. Moreover, demodex infection can aggravate ocular surface discomfort. Monitoring ocular surface health and treating demodex are crucial in prostate cancer patients taking ADT.

## Figures and Tables

**Table 1:** Ocular surface assessment and statistical results among the groups. Data are shown as the mean ± SD. *P* value was calculated using the Mann–Whitney *U* test.

	Ctrl	ADT	*P*	Asymptomatic ADT group	Symptomatic ADT group	*P*
Age OSDI	72.30 ± 6.19 8.31 ± 1.15	74.77 ± 8.62 11.72 ± 1.28	0.276 0.04	72.22 ± 7.88 -	75.61 ± 8.97 -	0.418 -
NI-BUT (s)	10.07 ± 4.05	7.60 ± 4.20	0.006	8.13 ± 4.27	5.32 ± 2.23	0.033
TMH(mm)	0.24 ± 0.08	0.21 ± 0.07	0.134	0.22 ± 0.07	0.19 ± 0.05	0.085
Lid margin abnormality score	1.10 ± 0.95	1.95 ± 0.79	≤0.001	2.02 ± 0.78	1.94 ± 0.80	0.666
Meibum expressibility	1.95 ± 0.90	2.53 ± 0.92	0.007	2.38 ± 0.93	2.94 ± 0.72	0.013
Meiboscores	1.05 ± 0.87	1.75 ± 0.81	≤0.001	1.57 ± 0.73	2.22 ± 0.80	0.005

**Table 2:** Demodex infection rate among the groups.

	Ctrl	ADT	Symptomatic ADT group	Asymptomatic ADT group
Demodex infection rate (%)	60.00%	70.00%	100%	57.14%
*X* ^2^	0.53		—	
*P*	0.46^a^		0.029^b^	

**Table 3:** Correlation between the duration of ADT and each ocular test. *P* value was calculated using Spearman's correlation test. *P* < 0.05 was considered statistically significant.

	NI-BUT (s)	TMH (mm)	Lid margin abnormality score	Meibum expressibility	Meiboscores	Time and ADT
NI-BUT (s)	RA					

TMH(mm)	*R* = −0.144	RA				
	*P* = 0.274					

Lid margin abnormality score	*R* = 0.076	*R* = −0.048	RA			
	*P* = 0.565	*P* = 0.713				

Meibum expressibility	*R* = 0.02	*R* = 0.067	*R* = 0.157	RA		
	*P* = 0.882	*P* = 0.611	*P* = 0.23			

Meiboscores	*R* = −0.116	*R* = 0.144	*R* = −0.016	*R* = 0.390^∗∗^	RA	
	*P* = 0.377	*P* = 0.272	*P* = 0.901	*P* = 0.002		

Time and ADT	*R* = −0.563^∗∗^	*R* = 0.161	*R* = −0.006	*R* = 0.304^∗^	*R* = 0.132	RA
	*P* ≤ 0.001	*P* = 0.22	*P* = 0.963	*P* = 0.018	*P* = 0.316	

## Data Availability

All data used to support the findings of this study is available on request.
